# Extremes of Interferon-Stimulated Gene Expression Associate with Worse Outcomes in the Acute Respiratory Distress Syndrome

**DOI:** 10.1371/journal.pone.0162490

**Published:** 2016-09-08

**Authors:** Jerry A. Nick, Silvia M. Caceres, Jennifer E. Kret, Katie R. Poch, Matthew Strand, Anna V. Faino, David P. Nichols, Milene T. Saavedra, Jennifer L. Taylor-Cousar, Mark W. Geraci, Ellen L. Burnham, Michael B. Fessler, Benjamin T. Suratt, Edward Abraham, Marc Moss, Kenneth C. Malcolm

**Affiliations:** 1 Department of Medicine, National Jewish Health, Denver, Colorado, United States of America; 2 Department of Pediatrics, National Jewish Health, Denver, Colorado, United States of America; 3 Division of Biostatistics and Bioinformatics, National Jewish Health, Denver, Colorado, United States of America; 4 Division of Pulmonary Science and Critical Care Medicine, Department of Medicine, University of Colorado Denver School of Medicine, Aurora, Colorado, United States of America; 5 St Louis County Department of Public Health, Berkeley, Missouri, United States of America; 6 Immunity, Inflammation, and Disease Laboratory, National Institute of Environmental Health Sciences, NIH, Research Triangle Park, North Carolina, United States of America; 7 Department of Medicine, University of Vermont College of Medicine, Burlington, Vermont, United States of America; 8 Office of the Dean, Wake Forest School of Medicine, Winston-Salem, North Carolina, United States of America; University of Tübingen, GERMANY

## Abstract

Acute Respiratory Distress Syndrome (ARDS) severity may be influenced by heterogeneity of neutrophil activation. Interferon-stimulated genes (ISG) are a broad gene family induced by Type I interferons, often as a response to viral infections, which evokes extensive immunomodulation. We tested the hypothesis that over- or under-expression of immunomodulatory ISG by neutrophils is associated with worse clinical outcomes in patients with ARDS. Genome-wide transcriptional profiles of circulating neutrophils isolated from patients with sepsis-induced ARDS (n = 31) and healthy controls (n = 19) were used to characterize ISG expression. Hierarchical clustering of expression identified 3 distinct subject groups with Low, Mid and High ISG expression. ISG accounting for the greatest variability in expression were identified (*MX1*, *IFIT1*, and *ISG15*) and used to analyze a prospective cohort at the Colorado ARDS Network site. One hundred twenty ARDS patients from four urban hospitals were enrolled within 72 hours of initiation of mechanical ventilation. Circulating neutrophils were isolated from patients and expression of ISG determined by PCR. Samples were stratified by standard deviation from the mean into High (n = 21), Mid, (n = 82) or Low (n = 17) ISG expression. Clinical outcomes were compared between patients with High or Low ISG expression to those with Mid-range expression. At enrollment, there were no differences in age, gender, co-existing medical conditions, or type of physiologic injury between cohorts. After adjusting for age, race, gender and BMI, patients with either High or Low ISG expression had significantly worse clinical outcomes than those in the Mid for number of 28-day ventilator- and ICU-free days (*P* = 0.0006 and 0.0004), as well as 90-day mortality and 90-day home with unassisted breathing (*P* = 0.02 and 0.004). These findings suggest extremes of ISG expression by circulating neutrophils from ARDS patients recovered early in the syndrome are associated with poorer clinical outcomes.

## Introduction

Extensive variability in severity and survival is a common feature of acute respiratory distress syndrome (ARDS), and identification of mechanisms that regulate this variability may lead to more personalized treatment. Age, race, cigarette smoking, and alcoholism impair the immune system, and are linked to increased prevalence or worse ARDS outcomes [1–9]. Specific forms of inflammatory dysregulation are also linked to worse outcomes from sepsis or ARDS, including coding variations in over 25 genes [10–12]. ARDS is most commonly precipitated by pneumonia or sepsis [9,13], resulting in massive neutrophil accumulation within the pulmonary vasculature [14]. Both over-exuberant or diminished innate immune response to bacterial products can worsen clinical outcomes, as the protective benefit of pathogen killing is balanced against the considerable injurious capacity of neutrophils [15,16]. Various stimuli may evoke complex “adaptive” responses to pathogens by neutrophils, by either decreasing (tolerance) or increasing (priming) activation [17,18]. Neutrophil function appears dysregulated in ARDS [19–26], and the potential exists that a beneficial adaptation to one microbe may place the host at a disadvantage against other infectious agents or inflammatory insults.

Viral infections can modify the immune response to subsequent bacterial infections [27–34], and thus could predispose to ARDS. A principal immune response to viral infections is production of the Type I interferons (IFNα and IFNβ), which are evoked by a broad range of viral factors, and in turn upregulate expression of interferon-stimulated genes (ISG) [35–39]. Hundreds of diverse ISG have been identified, with gene products which may act to reduce viral replication and release [40], or alternatively function as inflammatory cytokines [35,41,42]. However, ISG upregulation is neither sensitive nor specific for viral infection. Not all viral infections trigger the response, and certain intracellular bacteria or systemic autoimmune disorders have also been associated with IFNα/β release and ISG upregulation [35,43]. In animal models and human neutrophils, our group and others have reported elevated IFNα/β release and/or ISG expression is associated with impaired response to specific bacteria [43–48]. Conversely, severe bacterial-induced inflammation can suppress Type I IFN-regulated pathways, and certain viruses have acquired virulence factors that inhibit ISG [49–54].

Based on findings that ISG expression can modify the immune response, we hypothesized that both elevated and suppressed ISG expression could be associated with more severe outcomes in ARDS. Herein, we analyzed the range of neutrophil ISG expression in ARDS patients, and the correlation with clinical outcomes. Hierarchical clustering of neutrophil ISG demonstrated the presence of 3 distinct cohorts of subjects with High, Mid, or Low ISG expression. Three ISG with the greatest variability in expression were selected for expression analysis, and the association of these ISG expression levels with clinical outcome was prospectively tested at the onset of ARDS.

## Material and Methods

### Ethics Statement

The study protocol was approved by the Colorado Multiple Institutional Review Board (COMIRB) committee and the National Jewish Health Institutional Review Board. All subjects, or an appropriate proxy, gave written informed consent. The study was conducted in accordance with the Declaration of Helsinki.

### Enrollment of healthy controls

Volunteers (n = 40) were administered an 18-point questionnaire to verify they were not experiencing any acute symptoms of a viral illness, did not have any recently diagnosed medical problems, vaccinations, underlying chronic conditions, or had recently received blood products. In addition, these healthy subjects were verified to have negative screening results for hepatitis B and HIV within 6 months of the blood collection. Vital signs checked on the day of the collection were within the normal range.

### Subjects with ARDS used to characterize ISG

Peripheral blood neutrophils isolated from patients with sepsis-induced ARDS (n = 31) were used to establish ISG expression levels. Neutrophils were isolated within 24 hours of meeting criteria for ARDS criteria. Gene expression analysis from a subset of this cohort were previously reported in an analysis of HMGB1 and LPS induced patterns of gene expression in ARDS [55].

### Whole transcriptome analysis of neutrophil ISG expression to characterize ISG expression

Samples were obtained from 31 patients and 19 healthy volunteers. Briefly, total RNA was stabilized in freshly isolated neutrophils by resuspension of 2x10^7^ cells in 1 ml of RNAlater (Ambion, Austin, Texas), then stored at –20°C. Subsequent isolation with Trizol (Life Technologies, Rockville, Md.) and purification with RNEasy MinElute columns (Qiagen, Valencia, Calif.) was performed following the manufacturer’s protocol. Between 1 and 5 μg of total RNA was used for microarray target labeling using standard methods for reverse transcription and one round of *in vitro* transcription [56]. HG-U133A microarrays were hybridized with 10 μg cRNA and processed per the manufacturer’s protocol (Affymetrix, Foster City, Calif.). Experiments for this study were performed as recommended by the Microarray Gene Expression Data society [57]. Individual arrays were determined to be of high quality [58] by: (a) visual inspection; (b) comparison of the overall fluorescence intensity (scaling factor) to other arrays in the group; and (c) low 3’/5’ ratios for GAPDH and β-actin (ratio *<* 3). This procedure insures that each of the arrays in the group can be directly compared, and that the input mRNA was intact. Single channel hybridization was performed per sample. The internal quality control for each hybridization included comparison of overall intensity across all arrays (intensity consistently varied within two standard deviations of the median intensity) and the integrity of the labeled target as determined by the ratio of hybridization intensity to 3’ and 5’ regions of GAPD and β-actin (3’/5’ ratios were less than 3 for all arrays). RNA quality was assessed by spectrophotometry (A260/A280*>* 1.8) and Agilent Bioanalyzer (28S/18S rRNA intensity *>* 1.5). Affymetrix Eukaryotic Hybridization Control mixture was employed as external control. The complete set of gene expression data has been deposited in the GEO database (www.ncbi.nlm.nih.gov/geo/, accession #GSE3037).

### Analyses of gene expression in subjects to establish ISG variability

Gene expression analysis was performed using modules within MeV 4.6 [59]. Genes included in the “Type I Interferon-mediated Signaling Pathway Cluster” by the Gene Ontology Consortium (GO:0060337) were analyzed by Significance Analysis for Microarrays to eliminate genes whose expression did not vary between subjects and to detect those genes with significant variation between subjects. Hierarchical clustering was performed on this subset using Euclidean distance and complete linkage. Variability of gene expression was examined descriptively by comparing standard deviations, and principal Component Analysis was also performed on the genes using median centering. These analyses independently identified *Mx1*, *ISG15*, *IFIT1*, *and IFIT3* as genes whose expression differences were the greatest between subjects. A three-gene panel including *MX1*, *ISG15*, and *IFIT1* was used in subsequent analyses; *IFIT3* was eliminated from further analyses because of the high similarity, known co-expression, and co-evolutionary origin between it and *IFIT1*.

### Prospective evaluation of ISG expression from circulating neutrophils of ARDS patients

Circulating neutrophils of ARDS patients (n = 120) were isolated from patients enrolled into one of four NHLBI ARDS Network studies conducted at the University of Colorado affiliated hospitals, each with identical enrollment criteria. The parent studies were: Drug Study of Albuterol to Treat ALI (ALTA, ClinicalTrials.gov Identifier: NCT00434993)[60], Early Versus Delayed Enteral Feeding and Omega-3 Fatty Acid/Antioxidant Supplementation for Treating People With ALI or ARDS (EDEN-Omega Study: NCT00609180), or Early Versus Delayed Enteral Feeding to Treat People With ALI or ARDS (The EDEN Study: NCT00883948) [61,62], or Statins for Acutely Injured Lungs From Sepsis (SAILS: NCT00979121) [63]. The proportion of patients enrolled from each parent study was not different between the cohorts with High, Mid, or Low ISG expression (definitions for these groups described below)(**Table 1**). None of the interventions tested in the parent studies resulted in a treatment benefit [60–63]. The central inclusion criteria for all of these studies was ARDS, as defined by occurrence within a 24-hour time period of an acute onset of hypoxia (PaO_2_/FiO_2_<271 –adjusted for Denver altitude of 1600m), bilateral infiltrates by chest radiograph, and requirement for intubation and positive pressure ventilation in the absence of evidence for left-sided cardiac failure or other exclusionary criteria[60]. Sepsis was identified by standard criteria [64]. All patients enrolled into the parent ARDS Network studies were eligible for this ancillary study. All subjects were ventilated using the same protocol[60–62]. Peripheral blood samples were obtained 2.4±1.1 (mean±S.D.) days after ARDS criteria were met (2.6±1.2 days after intubation). Sample processing and data analysis was performed at NJH, with approval by the National Jewish Health Institutional Review Board. This ancillary trial was also registered in ClinicalTrials.gov (Identifier: NCT00548795). Diagnostic testing for acute or chronic viral infections was performed in the context of clinical care, and not as part of the study protocol. There were no common subjects between the two patient sets used in this study.

**Table 1:** ARDSNET study enrollment numbers (%) by ISG group.

Study	Mid-range ISG (n = 82)	High ISG (n = 21)	Low ISG (n = 17)	Overall *P*-value^a^
ALTA	14 (17.1)	5 (23.8)	3 (17.7)	*p* = 0.72
EDEN/OMEGA	58 (70.7)	15 (71.4)	14 (82.4)	*p* = 0.64
SAILS	14 (17.1)	1 (4.8)	0 (0)	*p* = 0.09

^a^P-value corresponds to Fisher’s exact test for categorical variables comparing three groups.

### Isolation of neutrophils and RNA from ARDS and healthy controls

Neutrophils were isolated from peripheral blood using the plasma Percoll method [65] in an identical fashion for healthy subjects and ARDS patients. Cells were confirmed to be >95% pure by visual inspection of cytospins. RNA was extracted immediately from 10–20 x 10^6^ isolated neutrophils in the absence of *ex vivo* stimulation using TRIzol reagent [66].

### PCR analysis of ISG expression

Gene expression in ARDS patients and healthy controls was quantified by real-time PCR of *MX1*, *IFIT1*, and *ISG15* relative to *GAPDH* by the ΔCt method using standard conditions. Primers and probes were obtained from Applied BioSystems (*MX1*, Hs00182073_m1; *ISG15*, Hs00192713_m1; *GAPDH* Endogenous Control) and Roche (*IFIT1*, Universal Probe Library #9 and forward: 5’-AGAACGGCTGCCTAATTTACA-3’; reverse: 5’-GCTCCAGACTATCCTTGACCT-3’) as previously described [48]. Relative ISG expression was multiplied by 10^5^ to convert all values greater than 1, and the mean of the log_2_ transformed expression values for each subject was calculated (S1 Dataset).

### Determination of IFNα levels

IFNα levels were measured from plasma of ARDS patients (n = 111) using an ELISA from PBL Laboratories, as directed by the manufacturer. Plasma was isolated during the course of neutrophil isolation.

### Data analysis of ISG groups

Because hierarchical clustering is less reliable for grouping samples when the number of variables is small, we divided groups based on deviation from the mean. Other methods to model high versus low ISG expression in ARDS patients were tested (sum of expression values, splitting into high, median and low ISG by quartiles, dichotomizing at the mean, dichotomizing at the median), but were found to have worse model fit by Akaike information criterion comparison (not shown). Overall group comparisons were made using ANOVA or Kruskal-Wallis tests for continuous variables and Fisher’s exact tests for categorical variables, as indicated in the text. Pairwise comparisons were made between the High and Mid-range and Low and Mid-range ISG groups when the corresponding overall test was significant. Ventilator-free and ICU-free days [67] were analyzed using linear regression. Twenty-eight day home with unassisted breathing and mortality and 90-day home with unassisted breathing and mortality were modeled using logistic regression. Cox proportional hazards models were used to model time to discharge to home and mortality outcomes. All regression and survival analyses were adjusted for gender, race, age and BMI. P-values corresponding to two-tailed tests that were less than 0.05 were considered statistically significant. Analyses were performed using SAS (Version 9.4, SAS Institute), and plotted using GraphPad Prism software.

## Results

### Transcriptional profiling of ISG expression in ARDS neutrophils

**Fig 1:**
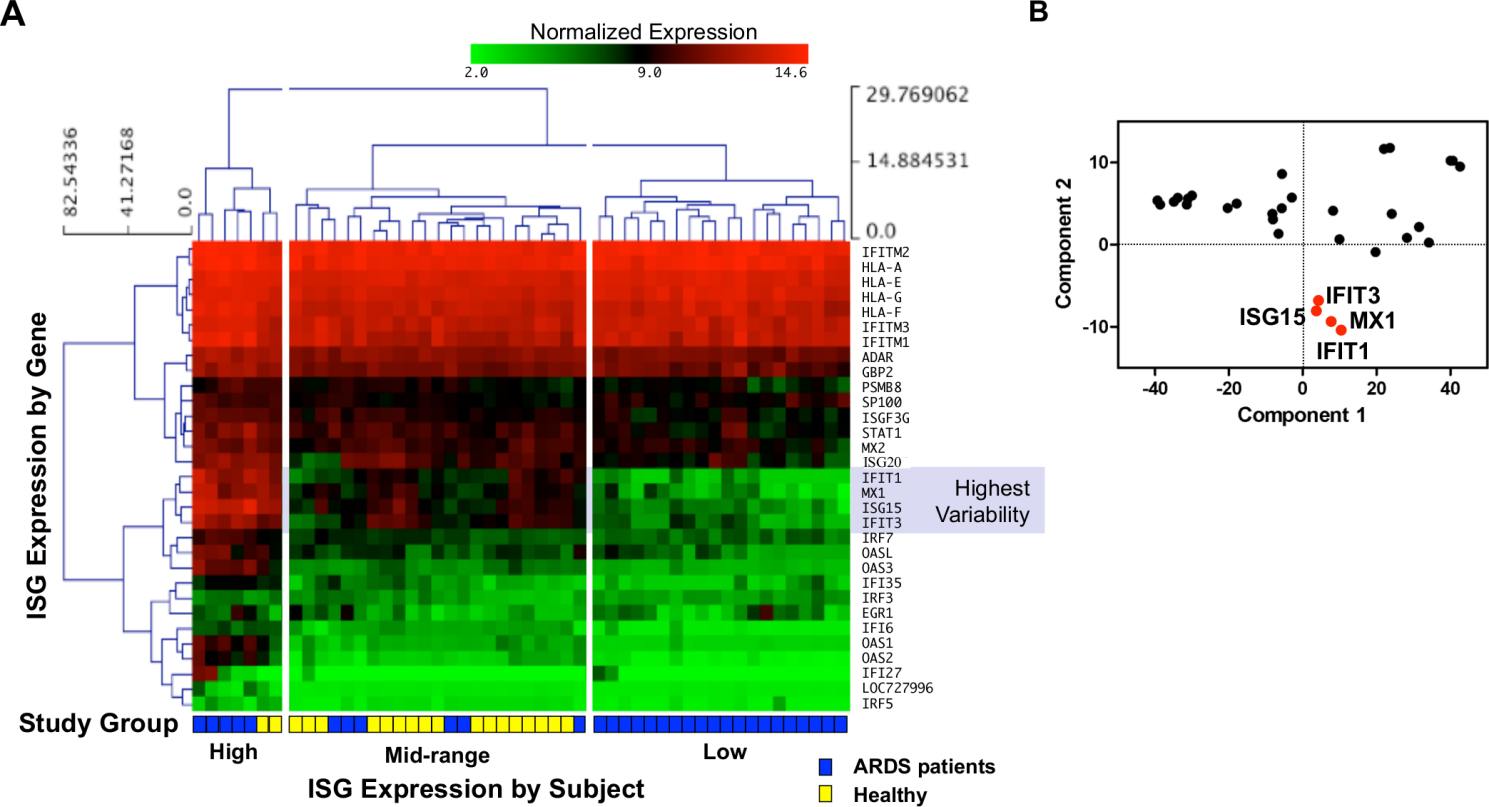
Transcriptome analysis to characterize ISG expression of ARDS patient and healthy control neutrophils. **(A)** Log_2_-transformed expression of 31 unique transcripts within the Type I Interferon-mediated Signaling Pathway Cluster from neutrophils isolated from sepsis-induced ARDS patients (n = 31) and healthy volunteers (n = 19). ISG expression was ordered by hierarchical clustering (Euclidean distance with complete linkage), and contains only genes determined to significantly change between subjects. Three major clusters of subjects (columns) are broadly grouped as High ISG expression (left), Mid (middle) and Low (right). Subject groupings are represented by yellow (healthy) and blue (ARDS) blocks at the profile base. Only ARDS patients were contained within the Low ISG expression subject cluster. When these genes were ranked for extent of variance of expression using relative size of the standard deviation between subjects, the genes with the largest variance were identified as *MX1*, *ISG15*, *IFIT1*, *and IFIT3* (identified by shading). **(B)** A Principal Component Analysis identified the same 4 genes that comprised nearly all of the variability between subjects in Panel A.

Genome-wide transcriptional profiles of circulating neutrophils isolated from patients with sepsis-induced ARDS (n = 31) and healthy controls (n = 19) was used to characterize ISG expression. Genes identified as the “Type I Interferon-mediated Signaling Pathway Cluster” by the Gene Ontology Consortium (GO:0060337) yielded 66 unique genes with available expression data. Significance Analysis for Microarrays determined that 31 genes were significantly changed between subjects. Using hierarchical clustering of expression, 3 distinct subject groups were identified. Both healthy controls and patients were found distributed in clusters of High and Mid ISG expression, while only ARDS patients were found within a cluster of Low ISG expression (**Fig 1**).

A test for variance was used to identify ISG that were representative of variability between subjects. When these genes were ranked for extent of variance of expression levels using relative size of the standard deviation between subjects, the genes with the largest variance were identified as *MX1*, *ISG15*, *IFIT1*, *and IFIT3* (**Fig 1A**). Alternatively, when gene expression was examined by Principal Component Analysis to discern changes in variation, the same four previously identified genes were identified as a distinct cluster (**Fig 1B**). PC1 (accounting for 91.6% of the variability) appears to generally represent average expression across subjects (high expression at the low end). PC2 (accounting for 4.5% of the variability) appears to distinguish within gene variability, where genes with the highest variability in expression are at the low end.

### ISG expression in neutrophils isolated from ARDS patients and healthy controls

**Fig 2:**
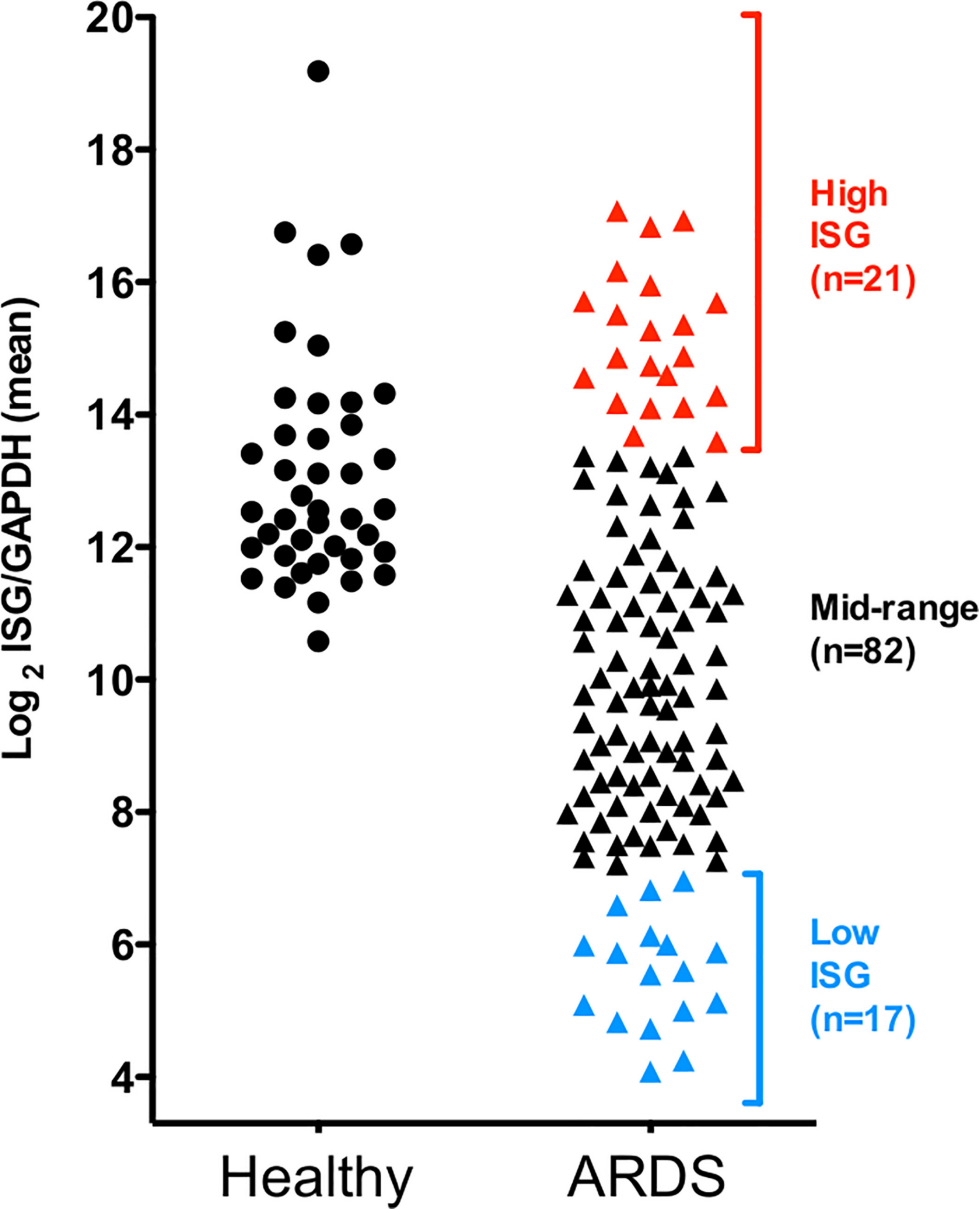
Classification of ARDS patients based on neutrophil ISG expression. **The mean of the** transformed expression values of neutrophil *MX1*, *IFIT1*, and *ISG15* is plotted for ARDS patients (triangle, n = 120) or healthy subjects (circle, n = 40). Subjects whose ISG expression was greater or less than one standard deviation from the mean were designated as High (red) or Low (blue) ISG expressers, respectively. ISG expression in healthy subjects overlapped only with the High and Mid cohorts of ARDS patients.

A three-gene panel (*MX1*, *ISG15*, and *IFIT1*) identified as having the most variable expression (**Fig 1**) was used as a marker of neutrophil ISG expression in ARDS. *IFIT3* was not further analyzed due to its high similarity, co-regulation, nearby chromosomal location, and co-evolutionary origin with IFIT1. We hypothesized that the variable expression of these genes would be associated with changes in clinical outcomes in ARDS. Relative expression level of each gene was determined prospectively by quantitative PCR of neutrophils isolated from ARDS patients (n = 120). The means of the transformed values of gene expression were normally distributed (**Fig 2**). Samples greater than one standard deviation above mean expression were designated as the “High” ISG expression cohort (n = 21), and the corresponding group of samples with ISG expression less than one standard deviation below the mean was designated as the “Low” ISG expression cohort (n = 17). Subjects falling within one standard deviation of the mean were designated as “Mid” ISG expression (n = 82) (**Fig 2**). ARDS patients in the test set were compared with healthy control subjects (n = 40) analyzed in an identical fashion. As with the first cohort, ISG expression of healthy controls was found to fall only within the High and Mid-ranges (**Fig 2**), while the Low-expressing cohort was confined to subjects with ARDS.

### Patient characteristics at study enrollment

Demographic features and underlying medical conditions for ARDS patients were quite similar across cohorts at the time of enrollment (**Table 2)**. Compared to the Mid cohort, patients with High and Low ISG expression were not different in age or gender. Within the High ISG cohort, 19% were identified as Black or African American, compared to 2.4% of the Mid cohort (pairwise *P* = 0.015). No differences in race or ethnicity were identified in the Low ISG cohort compared to Mid. BMI was significantly lower in the Low ISG cohort compared to the Mid cohort (pairwise *P* = 0.006). However, the Low ISG cohort did not have an increased frequency of identified pre-existing medical conditions. Patients with at least one identified underlying medical condition ranged from 63.4% to 71.4% across the three groups (Fisher’s test *P* = 0.80).

**Table 2:** Baseline characteristics of ARDS patients.

Characteristic	Mid-range ISG (n = 82)	High ISG (n = 21)	Low ISG (n = 17)	Overall *P*-value^a^
Male sex no. (%)	45 (54.9)	9 (42.9)	9 (52.9)	*P* = 0.67
Age in years (mean±S.D.)	53.7±16.2	53.4±11.7	55.1±14.2	*P* = 0.93
White race no. (%)	63 (76.8)	14 (66.7)	10 (58.8)	*P* = 0.23
Black or African American no. (%)	2 (2.4)	4 (19.0)	2 (11.8)	***P* = 0.01**^**b**^
Hispanic no. (%)	16 (19.5)	6 (28.6)	5 (29.4)	*P* = 0.45
BMI (mean±S.D.)	28.7±6.5	29.8±8.1	24.0±4.5	***P* = 0.02**^**c**^
Underlying Medical Conditions				
Any Medical Condition- no. (%)	52 (63.4)	15 (71.4)	11 (64.7)	*P* = 0.80
Chronic dialysis- no. (%)	1 (1.2)	1 (4.8)	2 (11.8)	*P* = 0.06
AIDS- no. (%)	2 (2.4)	2 (9.6)	0 (0)	*P* = 0.23
Leukemia- no. (%)	0 (0)	0 (0)	0 (0)	*P* = 1.0
Non-Hodgkin’s lymphoma- no. (%)	0 (0)	0 (0)	0 (0)	*P* = 1.0
Solid tumor w/ metastasis- no. (%)	2 (2.4)	1 (4.8)	0 (0)	*P* = 0.68
Immune suppression- no. (%)	5 (6.1)	4 (19.1)	0 (0)	*P* = 0.06
Hepatic failure- no. (%)	2 (2.4)	0 (0)	0 (0)	*P* = 1.0
Cirrhosis- no. (%)	8 (9.8)	3 (14.3)	2 (11.8)	*P* = 0.74
Diabetes Mellitus- no. (%)	16 (19.5)	5 (23.8)	7 (41.2)	*P* = 0.17
History of hypertension- no. (%)	29 (35.4)	8 (38.1)	6 (35.3)	*P* = 0.96
Prior myocardial infarction- no. (%)	5 (6.1)	1 (4.8)	2 (11.8)	*P* = 0.64
Peripheral vascular disease- no. (%)	3 (3.7)	1 (4.8)	1 (5.9)	*P* = 0.81
Prior stroke with sequelae- no. (%)	1 (1.2)	0 (0)	2 (11.8)	*P* = 0.07
Dementia- no. (%)	3 (3.7)	1 (4.8)	1 (5.9)	*P* = 0.56
Chronic pulmonary disease- no. (%)	9 (11.0)	2 (9.5)	0 (0)	*P* = 0.49
Arthritis- no. (%)	4 (4.9)	0 (0)	0 (0)	*P* = 0.77
Peptic ulcer disease- no. (%)	3 (3.7)	2 (9.5)	0 (0)	*P* = 0.34

^a^*P*-value corresponds to overall ANOVA for continuous variables comparing three groups, and Fisher’s exact test for categorical variables comparing three groups.

^b^ Significant difference between Mid-range and High ISG groups.

^c^ Significant difference between Mid-range and Low ISG groups.

Identified risk factors for ARDS at presentation were also not different between the study groups (**Table 3**). The most common presenting injury was pneumonia, with a prevalence ranging from 71.9% to 76.5% between the three groups (Fisher’s test *P* = 0.95), followed by secondary sepsis (range 57.1% to 73.2%, Fisher’s test *P* = 0.33). No patient had primary trauma. Study enrollment occurred 2.4±1.1 days (mean±S.D.) after meeting ARDS criteria. Clinical and physiologic assessment made at this early time point demonstrated a divergence in disease severity (**Table 4**). While gas exchange as measured by the PaO_2_ to FiO_2_ ratio was not different between the three groups, the Glasgow Coma score (GCS) and the APACHE III score differed significantly, with the Mid group averaging a less severe score. GCS represented the major contributor to the observed difference within the APACHE III score.

**Table 3:** Injury etiology at initial presentation by ISG group.

Primary physiological injury	Mid-range ISG (n = 82)	High ISG (n = 21)	Low ISG (n = 17)	Overall *P*-value^a^
Pneumonia–primary	59 (71.9)	16 (76.2)	13 (76.5)	*P* = 0.95
Pneumonia–secondary	8 (9.8)	1 (4.8)	2 (11.8)	*P* = 0.71
Aspiration–primary	6 (7.3)	1 (4.8)	2 (11.8)	*P* = 0.75
Aspiration–secondary	20 (24.4)	3 (14.3)	1 (5.9)	*P* = 0.18
Multiple transfusions–primary	3 (3.7)	0 (0)	0 (0)	*P* = 1.0
Multiple transfusions–secondary	5 (6.1)	1 (4.8)	0 (0)	*P* = 0.83
Sepsis–primary	10 (12.2)	3 (14.3)	2 (11.8)	*P* = 0.91
Sepsis–secondary	60 (73.2)	12 (57.1)	12 (70.6)	*P* = 0.33

^a^*P*-value corresponds to Fisher’s exact test for categorical variables comparing three groups.

**Table 4:** Physiologic assesment at enrollment^a^ by ISG group.

ASSESSMENT	Mid-range ISG (n = 82)	High ISG (n = 21)	Low ISG (n = 17)	Overall *P*-value^b^
Gas exchange				
PaO_2_/FiO_2_	107±40.8	104±45.1	128±53.1	*P* = 0.16
APACHE III score	87.5±22.8	106.2±20.4	100.1±38.8	***P* = 0.006**^**c**^
Glasgow Coma Score	8.3±2.8	5.7±2.6	8.0±3.1	***P* = 0.002**^**c**^

^a^Values measured 2.4 ± 1.1 days (mean ± S.D.) after ARDS criteria met.

^b^*P*-value corresponds to overall ANOVA for continuous variables comparing three groups.

^c^ Significant difference between Mid-range and High ISG groups.

### Extremes in ISG expression are associated with decreased survival and greater disease severity

Outcomes for patients with High or Low ISG expression were compared with the Mid ISG cohort. High ISG expression was associated with worse outcomes, with fewer ventilator-free and ICU-free days over the first 28 days than the Mid cohort (pairwise *P*-values = 0.006 and 0.009, respectively **Table 5**). The fraction of patients discharged to home with unassisted breathing was significantly lower at 90 days for High ISG compared to Mid (pairwise *P*-value = 0.02, **Table 5**). Mortality was higher by 90 days for High ISG, with 33.3% reported as dead prior to discharge to home with unassisted breathing for High ISG, compared to 12.2% for the Mid ISG group (pairwise *P*-value = 0.04).

**Table 5:** Summary Statistics for Clinical Outcomes. Elements in table are median (Q1-Q3) or number (percent) as indicated.

Characteristic	Mid-range ISG (n = 82)	High ISG (n = 21)	Low ISG (n = 17)	Overall *P*-value^a^
28-day free^b^				
Ventilation, median (Q1-Q3)	18 (4–22)	4 (0–18)	2 (0–18)	***P* = 0.006**^**c**^
ICU, mean, median (Q1-Q3)	17 (6–22)	1 (0–16)	1 (0–14)	***P* = 0.02**^**c**^
Home with unassisted breathing				
^2^Day 28, No. (%)	20 (24.4)	6 (28.6)	4 (23.5)	*P* = 0.95
^2^Day 90, No. (%)	65 (79.3)	11 (52.4)	9 (52.9)	***P* = 0.01**^**c**^
Mortality				
^2^Day 28, No. (%)	7 (8.5)	4 (19.1)	5 (29.4)	***P* = 0.04**^**c**^
^2^Day 90, No. (%)	10 (12.2)	7 (33.3)	6 (35.3)	***P* = 0.02**^**c**^

^a^*P*-value corresponds to overall Kruskal-Wallis test for 28-day free data comparing three groups, and Fisher’s exact test for categorical variables comparing three groups

^b^Number of days that patient is both alive and free of mechanical ventilation or ICU care for the first 28 days or first 90 days since start of mechanical ventilation or ICU care.

^c^Significant pairwise P-values between Mid and High ISG groups and Mid and Low ISG groups are stated in the text.

Patients with Low ISG expression also demonstrated worse outcomes, similar in severity to the High ISG expression cohort, with fewer ICU-free days over the first 28 days compared to the Mid cohort (pairwise *P*-value = 0.02, **Table 5**). The fraction of patients discharged to home with unassisted breathing was lower at 90 days for Low ISG compared to Mid (pairwise *P*-value = 0.03, **Table 5**). Mortality was significantly increased at 28 days (pairwise *P*-value = 0.03), and by 90 days, with 35.3% reported as dead prior to discharge to home with unassisted breathing for Low ISG (*P* = 0.03 compared to Mid, **Table 5**).

### Multivariate analysis of risk associated with extremes in ISG expression

After adjusting for gender, race, age and BMI, both High and Low ISG expression cohorts had significantly fewer days ventilation-free compared to the Mid ISG cohort (*P*-values = 0.002 for both comparisons, **Table 6**). Similarly, after adjusting for covariates, both High and Low ISG expression cohorts had significantly fewer days ICU-free compared to the Mid ISG cohort (*P*-value = 0.001 for both comparisons, **Table 6**). Multivariate analysis was not performed for APACHE III score, as it measures elements of disease severity likely associated with extremes in ISG expression.

**Table 6:** Estimated Differences And Odds Ratios For Adjusted Clinical Outcomes, Between High And Mid-Range Or Low- And Mid-Range ISG Groups. All models adjusted for age, race (African American or other), gender and BMI.

Characteristic	High ISG (n = 21)	Low ISG (n = 17)	Overall *P*-value
28-day free^a^			
Ventilation, mean difference (95% CI)	-7.00 (-11.38, -2.62)	-7.52 (-12.33, -2.71)	**0.0006**^**c**^
ICU, mean difference (95% CI)	-7.02 (-11.32, -2.72)	-7.71 (-12.43, -2.98)	**0.0004**^**c**^
Home with unassisted breathing ^b^			
28 Day Period, odds ratio (95% CI)	1.62 (0.52, 5.05)	0.94 (0.26, 3.45)	0.69
90 Day Period, odds ratio (95% CI)	0.19 (0.06, 0.63)	0.20 (0.06, 0.73)	**0.004**^**c**^
Mortality ^b^			
Day 28, odds ratio (95% CI)	2.83 (0.65, 12.33)	4.23 (0.94, 18.97)	0.12
Day 90, odds ratio (95% CI)	4.90 (1.37, 17.52)	4.29 (1.11, 16.59)	**0.02**^**c**^

^a^For 28-day free variables, mean differences are High or Low ISG group mean minus Mid-range ISG group mean; p-value corresponds to Type 3 analysis from linear regression model.

^b^For home with unassisted breathing and mortality models, Odds Ratio are odds for High or Low ISG group relative to odds for Mid-range ISG group; *P*-value corresponds to Type 3 analysis from logistic regression model.

^c^ Significant pairwise P-values between Mid and High ISG groups and Mid and Low ISG groups are stated in the text.

**Fig 3:**
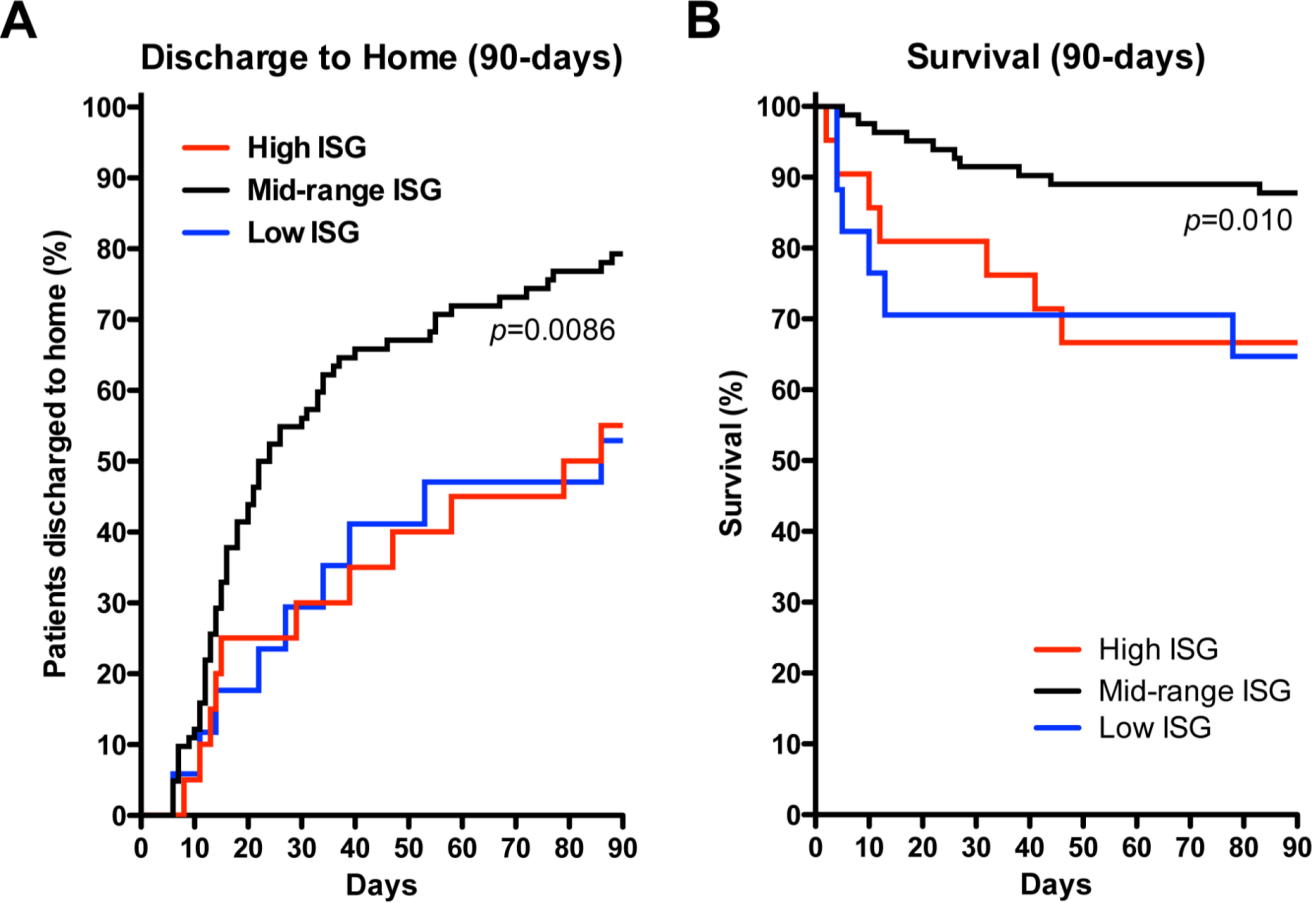
Kaplan-Meier Analysis of 90-Day Discharge to Home with Unassisted Breathing and Survival. **(A)** Proportion of patients confirmed to be discharged to home with unassisted breathing within 90 days of study enrollment. Overall log-rank *P*-value comparing groups was 0.009. After adjusting for age, race, gender and BMI, rate of discharge to home was significantly lower for both the High ISG (red line, *P* = 0.006) and Low ISG (blue line, *P* = 0.004; p-values from Cox PH model) expressing cohorts when compared to the Mid (black line). **(B)** Proportion of patients confirmed to be dead within 90 days of study enrollment. Overall log-rank *P*-value comparing groups was 0.01. After adjusting for age, race, gender and BMI, rate of death was significantly worse for both the High ISG (red line, *P* = 0.02) and Low ISG (blue line, *P* = 0.02; p-values from Cox PH model) expressing cohorts when compared to the Mid (black line).

Patients’ status of being discharged home with unassisted breathing by day 90 was significantly different for High and Low ISG expression cohorts, compared to the Mid cohort (*P*-values = 0.006 and 0.01, respectively, **Table 6**). Patients’ 90 day mortality was significantly different for High and Low ISG expression cohorts, compared to the Mid cohort (p-values = 0.01 and 0.035, respectively, **Table 6**). In addition, time to discharge to home with unassisted breathing was significantly delayed in the High and Low cohorts compared to the Mid cohort (*P*-values = 0.006 and 0.004, respectively; p-values from Cox PH model)(**Fig 3A**). A survival analysis of time to mortality by day 90 was also significantly different for High and Low ISG expression cohorts, compared to the Mid cohort (*P*-values = 0.01 and 0.009, respectively, from Kaplan Meier analysis; adjusted *P*-values = 0.02 and 0.02, respectively, from Cox PH model) (**Fig 3B**).

### Association of ISG expression with viral infection or circulating IFNα

**Fig 4:**
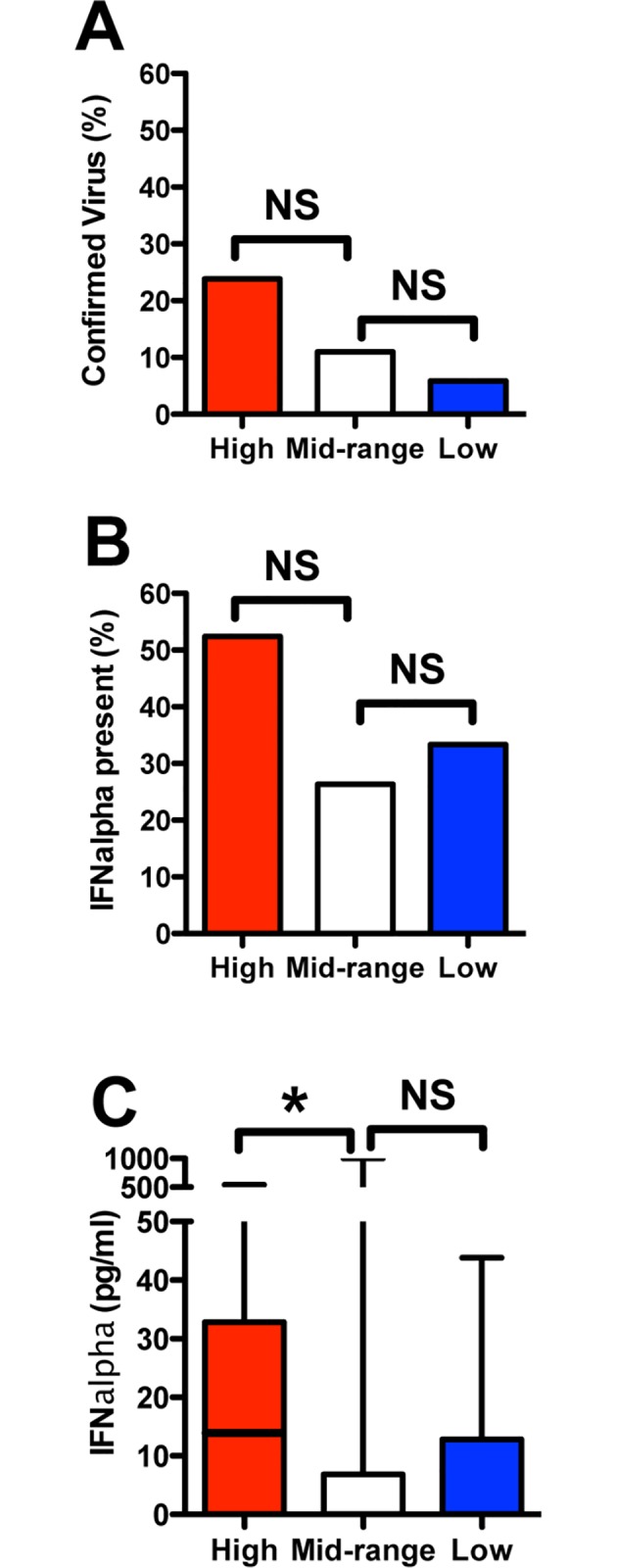
Association of ISG expression with confirmed viral infections and circulating IFNα. **(A**) The presence of a viral infection, as determined either through available medical history or within the course of clinical care, did not reach significance in the cohort with High ISG expression (5 of 21) compared to the Mid (8 of 82) and Low (1 of 17) cohorts (Fisher’s exact test *P* = 0.23). **(B)** Detection of circulating IFNα tended to occur more frequently within the High ISG cohort (11 of 21) compared to the Mid (20 of 76) and Low (5 of 15) cohorts, but this difference did not reach significance (Fisher’s exact test *P* = 0.08). **(C)** Levels of circulating IFNα trended higher within the High ISG cohort (n = 21) compared to the Mid (n = 76), (Kruskal-Wallis overall *P*-value = 0.03; High vs. Mid pairwise *P* = 0.009). n = 15 for the Low cohort. Plot depicts range (minimum≈0, maximum = upper whisker), 1^st^ to 3^rd^ quartile (box) and median (line).

Screening of patients for viral infection was not systemically performed. However, acute or chronic viral infections were identified in 15 subjects during clinical care, including H1N1 influenza (n = 6), HIV (n = 4), Hepatitis B (n = 1), Hepatitis C (n = 3), or H1N1 combined with Hepatitis C (n = 1). While 24% of the High ISG expression cohort had a confirmed viral infection, compared to 11% in the Mid ISG cohort and 5.9% in the Low ISG cohort, these differences were not significant (Fisher’s exact test *P* = 0.23)(**Fig 4**). We measured serum IFNα in patients as a possible surrogate to detect viral infection. At enrollment into this protocol, 52.4% of patients in the High ISG cohort had detectable IFNα in their circulation, which trended greater than patients in the Mid ISG cohort (26.3%) and the Low ISG cohort (33.3%, Fisher’s exact test *P* = 0.08)(**Fig 4**). The quantity of IFNα in circulation was highly variable, but greater in the High ISG cohort (median 13.3 pg/mL, range <0.1 to 544) compared to the Mid ISG cohort (median <0.1, range <0.1 to 1000, Kruskal-Wallis *P* = 0.009)(**Fig 4**).

## Discussion

This single-center prospective study is the first known exploration of the clinical significance of systemic ISG expression in ARDS. In a preliminary set of patients (n = 31) we identified the ISGs that account for the greatest variability in expression. These genes (*MX1*, *ISG15*, *IFIT1*, *and IFIT3*) are prototypical ISGs, previously shown to be highly expressed in stimulated neutrophils [66,68,69], lymphocytes [37], peripheral blood mononuclear cells [38,39], and whole blood [70]. Using a panel of three of these unrelated genes in a non-overlapping cohort (n = 120), we found that ARDS patients with ISG expression greater or less than one standard deviation from the mean had significantly worse clinical outcomes. These data suggest the potential for immunomodulation as a result of ISG expression to affect ARDS outcomes. At the time of admission, these three cohorts were indistinguishable with regards to age, gender, co-existing medical conditions, or type of physiologic injury. Likewise, circulating white blood cell count or plasma levels of IL6 and TNFα showed no correlation with ISG levels (data not shown). Use of single genes over the 3-gene panel offered no distinction in the analyses.

Occurrence of elevated ISG expression in a subgroup of ARDS patients and healthy controls is not surprising. Acute and chronic viral infections are common within the population [52], and may remain undiagnosed or indolent. While a higher prevalence of acute or chronic viral infection were identified in the High ISG group of ARDS patients (**Fig 4**), most of the High ISG group were not known to have a co-existing viral infection. Conversely, patients with proven viral infections were also found (in lower percentages) in the Mid and Low ISG expression cohorts, underscoring the relatively poor correlation between clinically evident viral infections and systemic ISG expression. Although IFNα/β and ISG expression is indicative for viral infections, we have no evidence outside the confirmed cases that viral infection was responsible for ISG expression. IFNα/β is released into the circulation early after viral infections, and may be undetectable or present in very low quantities, even in experimental settings [36], a finding confirmed in this study. Thus direct testing for IFNα/β in the serum is not clinically useful. However, leukocyte ISG expression has been identified as a more sensitive marker of Type 1 IFN release [36]. ISG expression signatures have been proposed as a useful marker to distinguish between bacterial and viral infections [38], and even between specific viruses [39] as part of a paradigm shift to focus on host gene profiles to diagnosis infections rather than the traditional search for the pathogen [71]. Similarly, TLR4 activated IFNα/β production has been reported in human macrophages [72], and a sizeable subpopulation within the ARDS cohort were septic, suggesting that TLR4 activation could also be responsible for enhanced ISG expression. However, it is unknown if TLR4 mediates IFNα/β production by systemic cells, and ISG expression is equally elevated in some healthy subjects, suggesting that sepsis is not the major cause of ISG expression.

The association of worse clinical outcomes with High ISG expression in ARDS is supported by clinical observations and animal models that demonstrate viral infections increase susceptibility to secondary bacterial infections [27–34]. Primary or secondary bacterial infections precipitate an overwhelming majority of ARDS cases [10]. Previously, we reported that ARDS neutrophils with elevated ISG expression have an altered response to *S*. *aureus*, which included reduced p38 MAP kinase activation, attenuation of O_2_^-^ generation and IL8 secretion, increased apoptosis, and impaired bacterial killing [48]. Others have reported IFNα/β release and ISGs expression promote anti-bacterial activity against select pathogens [44–47]. No demographic or clinical feature at study enrollment associated with worse outcomes in the High ISG expression compared with the Mid group, with the exception of a greater prevalence of African Americans in the High ISG group (19% *vs* 2.4%, *P* = 0.015). African Americans have been found to experience worse outcomes from ARDS [8]. However, multivariate analysis confirmed that ISG expression was still a risk factor after accounting for race in the High expression cohort.

The presence of a distinct subpopulation of ARDS patients with relatively inhibited ISG expression was seen in both cohorts of patients used in this study. Outcomes in this cohort were also worse than the Mid ISG group, without differences in demographic or clinical features. The Low ISG group had a normal BMI, which was significantly lower than the Mid group, who were on average overweight (mean±sd: 24.0±4.5 vs 28.7±6.5, *P* = 0.006). However, the prevalence of pre-existing medical conditions was not greater in the Low ISG subgroup, and differences in BMI within this range are not associated with changes in outcomes from ARDS [73]. Worse outcomes in ARDS patients with abnormally Low ISG expression is supported by a preponderance of data that indicate ISG expression is generally a protective mechanism[41]. In the absence of ISG-encoded products, the host is more vulnerable to sustained or recurrent viral infection[74]. While ISG expression is believed to be induced by anti-viral signals, it is of interest that the Low ISG cohort has levels of some ISG that are lower than in healthy subjects, particularly the genes identified as having the greatest expression variability. Increasingly, viruses have been identified with the capacity to suppress ISG expression through a variety of mechanisms, including strains of herpes simplex virus I [50], rhinovirus [51], hepatitis C [52,54] and pseudorabies virus [49]. A recent trial demonstrated a reduction in ARDS mortality associated with the administration of IFN-beta-1a[75], supporting our finding that a subpopulation of ARDS patients with high mortality has low ISG expression.

In conclusion, systemic ISG expression within the first days of ARDS onset is associated with disease severity and prognosis. This response should be considered along with other identified genetic, environmental, and complex demographic factors as a contributor to heterogeneity of ARDS outcomes. Other identified disease modifiers, such as age, race, or alcoholism [1–9], or coding variation in inflammatory response regulating genes [10–12], are generally chronic or permanent risk factors. Yet no risk factor is apparent in many ARDS patients, and the general lack of recurrent ARDS argues for the existence of transient risk factor(s). Up or downregulation in ISG expression could represents a “window” of vulnerability that places an otherwise healthy subject at increased risk for a period of days or weeks. This is a clinically plausible scenario in many patients, supported by analysis of mortality from respiratory failure during viral pandemics [28,29]. While ISG expression was assessed early after clinical presentation in this trial, cytomegalovirus reactivation may occur 1–2 weeks after hospitalization in critically ill patients [76], which could upregulate ISG expression later in ARDS. Longitudinal studies are needed to determine the duration of abnormal ISG expression in ARDS patients, and if late upregulation occurs with CMV reactivation. While ISG expression may be of prognostic value at the onset of ARDS, the potential exists for this marker to modify clinical care, either by alerting clinicians to the possibility of an unsuspected viral or autoimmune disease, or as a direct target for immunomodulation through administration of Type 1 interferons[75].

## Supporting Information

S1 Dataset ISG expression in ARDS and healthy neutrophils. (TXT)
